# Ambiguity and uncertainty tolerance, need for cognition, and their association with stress. A study among Italian practicing physicians

**DOI:** 10.1080/10872981.2016.1270009

**Published:** 2017-01-09

**Authors:** Paola Iannello, Anna Mottini, Simone Tirelli, Silvia Riva, Alessandro Antonietti

**Affiliations:** ^a^ Department of Psychology – Research Unit on Decision-making Processes in Emergency Medicine, Catholic University of the Sacred Heart, Milan, Italy; ^b^ University of Milan, Department of Health Sciences, Milan, Italy

**Keywords:** Tolerance for ambiguity, uncertainty, need for cognitive closure, work-related stress, medical expertise, practicing physicians

## Abstract

Medical practice is inherently ambiguous and uncertain. The physicians’ ability to tolerate ambiguity and uncertainty has been proved to have a great impact on clinical practice. The primary aim of the present study was to test the hypothesis that higher degree of physicians’ ambiguity and uncertainty intolerance and higher need for cognitive closure will predict higher work stress. Two hundred and twelve physicians (mean age = 42.94 years; SD = 10.72) from different medical specialties with different levels of expertise were administered a set of questionnaires measuring perceived levels of work-related stress, individual ability to tolerate ambiguity, stress deriving from uncertainty, and personal need for cognitive closure. A linear regression analysis was performed to examine which variables predict the perceived level of stress. The regression model was statistically significant [R^2^ = .32; F(10,206) = 8.78, p ≤ .001], thus showing that, after controlling for gender and medical specialty, ambiguity and uncertainty tolerance, decisiveness (a dimension included in need for closure), and the years of practice were significant predictors of perceived work-related stress. Findings from the present study have some implications for medical education. Given the great impact that the individual ability to tolerate ambiguity and uncertainty has on the physicians’ level of perceived work-related stress, it would be worth paying particular attention to such a skill in medical education settings. It would be crucial to introduce or to empower educational tools and strategies that could increase medical students’ ability to tolerate ambiguity and uncertainty.

**Abbreviations:** JSQ: Job stress questionnaire; NFCS: Need for cognitive closure scale; PRU: Physicians’ reactions to uncertainty; TFA: Tolerance for ambiguity

## Introduction

Beliefs about the absolute certainty and truth of medical knowledge have been found to be quite common not only among laypeople [1], but also among medical students [2]. However, along the process of professional identity formation, most students develop an implicit belief about the uncertainty of medical knowledge [3] that modify their former understanding that medicine deals with black-and-white decisions. When confronted with actual medical practice, they realize that uncertainty and ambiguity surround every aspect of medicine. Limited medical knowledge when making a diagnosis or opting for a specific treatment [4], unpredictability of the natural course of illness, and variability of patients’ responses to treatment [5] may all represent potential sources of medical uncertainty.

Since uncertainty and ambiguity are pervasive in medical practice, physicians are constantly called to exercise judgment and decisions in ill-defined contexts and situations [6,7]. This situational uncertainty requires physicians the ability to properly react to it [8]. Literature consistently indicates that the capacity to tolerate and manage ambiguity and uncertainty represents a fundamental competence for physicians [2,9–11]. In health care, a number of studies found some correlations between physicians’ individual ability to tolerate ambiguity and their level of psychological well-being. In particular, physicians with low tolerance of ambiguity and uncertainty tend to report higher rate of referrals, burnout, and anxiety and lower level of satisfaction [10,12], less comfort in dealing with dying patients, and higher levels of dogmatism, rigidity, and conformism [13,14]. Additionally, low tolerance for ambiguity turned out to be associated with the tendency to order more diagnostic tests [15,16], with a negative impact on the costs for the health care system. Finally, some studies [17,18] suggested that different levels of tolerance for ambiguity could be considered as a predictor for the future specialty choice among medical students. Higher levels of ambiguity tolerance were associated with a preference for cognitive disciplines, which mainly rely on the integration of laboratory tests and anamnestic and clinical data to identify optimal management strategies (e.g., Internal medicine), whereas higher levels of ambiguity intolerance predicted the choice for procedural specialties, which require physicians to highly rely on procedure (e.g., Surgery).

Although often deemed as interchangeable, intolerance for ambiguity and intolerance for uncertainty correspond to slightly different constructs [19]. *Ambiguity* intolerance has been defined as the tendency to perceive ambiguous situations as a potential source of threat, referring to ambiguous stimuli as those that are characterized by novelty, complexity, and insolubility [20]. Whereas ambiguity intolerance concerns the (in)capacity to deal with equivocal situations in the present time, the intolerance for *uncertainty* can be described as the tendency to consider as unacceptable the possible occurrence of a future negative event [19,21]. Even though both constructs imply negative reactions, such as discomfort and uneasiness to equivocal stimuli, individuals who are high ambiguity intolerant tend to be threatened by an ambiguous stimulus in the ‘here and now’, whereas individuals who are high uncertainty intolerant feel threatened by an uncertain future stimulus and focus on the anticipation of possible future consequences [22].

The tolerance for ambiguity and the tolerance for uncertainty present some theoretical overlaps with the need for cognitive closure, which has been defined along a motivational continuum as ‘an individual’s desire for a firm answer on a given topic as compared to confusion and ambiguity’ [23]. Even though some situational elements, such as time pressure, tend to systematically increase the desire for closure, this aspect differs consistently across people. Specifically, individuals high in need for closure strive for closure, seek definite options, tend to be impatient in finding an answer, and are impulsive in judging and decision making [24]. Although ambiguity and uncertainty intolerance and need for cognitive closure are somehow correlated [25], their overlapping is only partial. To be more precise, the need for cognition identifies the motivation to approach or avoid cognitive closure, whereas in ambiguity and uncertainty intolerance the focus is on the psychological effects of uncertainty, namely, the individual’s negative reactions to equivocal stimuli [26].

Literature is quite relevant and rich [11] if we focus on the conceptual and theoretical issues associated with ambiguity and uncertainty tolerance, whereas little empirical research has been conducted on this topic. More precisely, the few empirical studies mainly involved medical students or registrars who were still in training [10,27,28]. Moreover, to our knowledge, no study considering the relationships among ambiguity, uncertainty intolerance, and need of cognitive closure was conducted. Lastly, no study examining the effect of these three variables together on the level of perceived stress is available.

## Purpose

The primary aim of the present study was to investigate the role of physicians’ intolerance of ambiguity and uncertainty and need for cognitive closure in predicting their level of perceived stress. We hypothesized that higher degree of ambiguity and uncertainty intolerance and higher need for cognitive closure will predict higher work-related stress.

The study included practicing physicians with different levels of expertise and with different medical specializations to control for these professional characteristics. As for the medical specialties, in the present study we referred to the broad distinction between cognitive and procedural specialties [29,30]. Cognitive disciplines are those which mainly rely on the integration of laboratory tests, anamnestic data, and other clinical information to make diagnostic and therapeutic decisions (e.g., Pediatrics), whereas procedural disciplines require physicians to be mostly concerned with decisions and outcomes connected to surgery [29,30].

## Methods

### Participants

Participants were recruited from 11 different hospitals located in Northern Italy. It is worth noting that all hospitals, which were situated in an area with similar geographic and demographic features, belonged to the national health care system (none of them are private), and therefore they used the same rules and protocols.

The sample included 212 practicing physicians. Table 1 reports the detailed description of participants in relation to age, gender, years of experience, and medical specialties (cognitive vs. procedural).

**Table 1.** Descriptive data of the sample.

Age		N = 212; range 26–67; Mean age = 42.9 years, SD = 10.7)
**Gender**	***Male***	N = 109 (51% of the total sample; Mean
		age = 46.9, SD = 10.5)
	***Female***	N = 103 (49% of the total sample; Mean
		age = 38.1, SD = 9.2)
**Years of experience**	***< 10***	N = 89 (Mean age = 33.3, SD = 4.2)
		(Male: N = 31; Female: N = 58)
	***10–20***	N = 56 (Mean age = 42.7, SD = 3.7)
		(Male: N = 28; Female: N = 28)
	***> 20***	N = 67 (Mean age = 56.1; SD = 4.8)
		(Male: N = 50; Female: N = 17)
Specialty*	***Cognitive***	N = 92 (Mean age = 45.9, SD = 5.4)
		(Male: N = 40; Female: N = 52)
	***Procedural***	N = 120 (Mean age = 40.3, SD = 6.4)
		(Male: N = 69; Female: N = 51)

* Physicians practiced18 different specialties, which were grouped into cognitive and procedural specialties as follows:

**Procedural**: General surgery (20%), Gastroenterology (8%), Anesthesiology (8%), Nephrology (5%), Orthopedic surgery (5%), Otolaryngology (4%), Emergency medicine (4%), Urology (2%), Gynecology (2%), Cardiothoracic surgery (1%),

**Cognitive**: Internal medicine (10%), Medical oncology (8%), Pediatrics (8%), Geriatric internal medicine (7%), Infectious diseases (4%), Physical medicine and rehabilitation (2%), Endocrinology(1%), Dermatology (1%)

### Materials

#### Tolerance for ambiguity (TFA) scale [31]

The seven-item TFA scale is a measure of one’s ability to cope with situations of ambiguity (e.g., ‘A good task is one in which what is to be done and how it is to be done are always clear’). The scale was originally tested on a population of physicians, even though it aims at measuring ambiguity tolerance as a general personal trait. For each item participants are required to indicate the extent to which they agree with the statement on a 6-point Likert scale. TFA scores are calculated by summing across the seven items. Higher TFA scores correspond to higher tolerance for ambiguity. We translated the scale into Italian using a standardized procedure including two independent translations, selection of a consensus translation by an expert panel, and pretest among 15 physicians. The translated scale demonstrated good levels of internal consistency for the present study (Cronbach’s alpha = .76)

#### Physicians’ reactions to uncertainty (PRU) [32]

The PRU is aimed at measuring physicians’ affective reactions to uncertainty in patient care. It consists of two subscales: the Stress from Uncertainty (SUS) subscale, which concerns anxiety and discomfort resulting from uncertainty (e.g., ‘The uncertainty of patient care often troubles me’), and the Reluctance to Disclose Uncertainty (RDU) subscale, which regards a fear for disclosing uncertainty (and possible bad outcomes of uncertainty) to others (e.g., ‘I almost never tell other physicians about diagnoses I have missed’). The RDU subscale mainly concerns a relational dimension within the management of uncertainty, which was beyond the scope of the present study. Given its relevance to the present study, the SUS subscale was included. In the 13-item SUS each item is scored on a 6-point Likert scale. We translated the scale into Italian using a standardized procedure including two independent translations, selection of a consensus translation by an expert panel, and pretest among 15 physicians. The internal consistency of the translated scale was similar to that of the original scale (Cronbach’s alpha = .83).

#### Need for cognitive closure scale [33] (NFCS; Italian validated version [34])

The NFCS assesses the extent to which a person expresses a need for definite answers, order, and closure and an aversion toward ambiguity. People with a high need for cognitive closure tend to strive for closure by avoiding new information and thus quickly reaching closure. They generally experience an urgent desire to obtain stable knowledge. The 42-item scale consists of five subscales: (1) Preference for order and structure (e.g., ‘I like to have a plan for everything and a place for everything’) (Cronbach’s alpha = .73); (2) Preference for predictability (e.g., ‘I do not like to go into a situation without knowing what can I expect from it’) (Cronbach’s alpha = .76); (3) Decisiveness (e.g., ‘I usually make important decisions quickly and confidently’) (Cronbach’s alpha = .70); (4) Discomfort with ambiguity (e.g., ‘I don’t like situations that are uncertain’) (Cronbach’s alpha = .69); (5) Close-mindedness (e.g., ‘I do not usually consult many different opinions before forming my own view’) (Cronbach’s alpha = .69). We used the Italian version of the NFCS [32]. A high level of internal consistency was observed also for the total scale (overall Cronbach’s alpha = .85).

#### Job stress questionnaire (JSQ) [35]

We used a version of JSQ, which is a modification of the original JSQ developed by Caplan and colleagues [36]. The scale consists of 10 items aimed at measuring the level of perceived stress associated with workload, role ambiguity, and utilization of skills. (e.g., ‘If I have more time, I would do my job in a better way’). The JSQ, which is short and easy to administer, shows good psychometric properties and is one of the most used scale to measure work-related stress. In our study the scale demonstrated a very high level of internal consistency (Cronbach’s alpha = .95).

### Procedure

Following ethical approval from the Ethical Committee of the Department of Psychology (Catholic University of the Sacred Heart) and after obtaining the authorization from the directors of all hospitals, physicians were initially approached by email and the purpose of the study was explained. Following this, the research team visited each hospital and handed out paper versions of the questionnaires. Physicians who voluntarily accepted to take part in the research were then asked to fill in the questionnaires. Approximately 30 minutes were required to complete the questionnaires. Physicians were given the possibility to fill in the questionnaires within five days. Following five days, the research team returned and collected the completed questionnaires.

### Data analyses

Data were analyzed using SPSS version 21.0 software (SPSS Inc., Chicago, USA).

First, descriptive statistics were calculated for each scale. Afterwards we verified the effect of the demographic and professional characteristics on all measures. We carried out a MANOVA to compare the levels of perceived work stress, ambiguity tolerance, stress from uncertainty, and need for cognitive closure depending on gender, years of experience, and specialty. Next, we examined the relationships between the level of perceived stress and all other measures using Pearson’s two-tailed correlations. Finally, a linear regression analysis was computed to examine the role of ambiguity tolerance, stress from uncertainty, and need for closure in predicting the perceived level of stress, by controlling for demographic and professional characteristics.

## Results

### Role of demographic and professional characteristics on level of perceived stress and tolerance of ambiguity, stress from uncertainty, need for cognitive closure

Descriptive statistics are reported in Table 2.

**Table 2.** Descriptive statistics for all measures.

	*JSQ*	*TFA*	*PRU* *stress from uncertainty*	*NFCS* *preference for order*	*NFCS* *preference for predictability*	*NFCS* *decisiveness*	*NFCS* *discomfort with ambiguity*	*NFCS* *closed-mindedness*
	*M (SD)*	*M (SD)*	*M (SD)*	*M (SD)*	*M (SD)*	*M (SD)*	*M (SD)*	*M (SD)*
Overall sample (N = 212)	2.62 (0.48)	25.40 (4.56)	43.87 (8.36)	4.16 (0.64)	3.91 (0.58)	4.29 (0.51)	3.93 (0.56)	3.16 (0.48)
Gender Women (N = 103) Men (N = 109)								
	2.71 (0.77)	25.64 (4.41)	45.21 (9.09)	4.11 (0.62)	3.82 (0.61)	4.24 (0.49)	3.90 (0.52)	3.07 (0.50)
	2.63 (0.78)	25.17 (4.72)	42.58 (7.45)	4.21 (0.65)	3.99 (0.54)	4.33 (0.52)	3.96 (0.59)	3.25 (0.44)
Years of experience < 10 (N = 89) 10–20 (N = 56) > 20 (N = 67)								
	2.79 (0.99)	25.72 (4.46)	45.84 (9.12)	4.17 (0.57)	3.86 (0.60)	4.26 (0.50)	3.85 (0.49)	3.08 (0.47)
	2.67 (0.83)	26.34 (4.34)	42.26 (7.74)	4.03 (0.66)	3.82 (0.54)	4.27 (0.49)	3.88 (0.58)	3.08 (0.43)
	2.53 (1.04)	24.25 (4.76)	42.92 (7.84)	4.28 (0.69)	4.04 (0.58)	4.33 (0.54)	4.05 (0.61)	3.34 (0.51)
Specialty Cognitive (N = 92) Procedural (N = 120)								
	2.66 (0.91)	25.36 (4.56)	42.97 (7.22)	4.10 (0.51)	3.84 (0.61)	4.31 (0.43)	3.91 (0.54)	3.25 (0.39)
	2.68 (0.62)	24.99 (4.67)	43.96 (8.41)	4.22 (0.64)	3.99 (0.52)	4.32 (0.57)	3.92 (0.53)	3.12 (0.50)

Mean values and standard deviations are presented for the overall sample and for each subgroup related to gender (men vs. women), years of experience (<10 vs. 10 to 20 vs. >20), and specialty (cognitive vs. procedural).

The results of the MANOVA are reported as follows.

#### Gender

For all dependent variables no significant differences emerged between male and female physicians (Wilks’s lambda = 0.95, p = .23: Level of perceived stress F(1,195) = 0.13, p = .71; Tolerance for ambiguity F(1,195) = 0.27, p = .87; Stress form uncertainty F(1,195) = 1.16, p = .28; Preference for order F(1,195) = 0.21, p = .65; Preference for predictability F(1,195) = 0.08 p = .78; Decisiveness F(1, 195) = 0.23, p = .63; Discomfort with ambiguity F(1,195) = 1.84, p = .18; Closed-mindedness F(1, 195) = 3.10, p = .06).

#### Years of experience

No significant differences emerged in relation to the level of physicians’ expertise (Wilks’s lambda = 0.91, p = .41: Tolerance for ambiguity F(2,195) = 1.29, p = .27; Stress from uncertainty F(2,195) = 0.71, p = .49; Preference for order F(2,195) = 0.41, p = .63; Preference for predictability F(2,195) = 0.24 p = .79; Decisiveness F(2, 195) = 0.15, p = .98; Discomfort with ambiguity F(2,195) = 0.14, p = .86; Closed-mindedness F(2, 195) = 0.77, p = .46), with the exception of the level of perceived stress (F(2,195) = 4.81, p < .05). Specifically, physicians with longer work experience (> 20 years) reported the lowest level of stress as compared to those physicians with less than 10 years of experience (Tukey’s test: p < .05).

#### Medical specialty

No significant differences between cognitive vs. procedural specialists emerged (Wilks’s lambda = 0.93, p = .08: Level of perceived stress F(1,195) = 0.13, p = .71; Tolerance for ambiguity F(1,195) = 3.80, p = .09; Stress from uncertainty F(1,195) = 1.76, p = .19; Preference for order F(1,195) = 0.47, p = .49; Preference for predictability F(1,195) = 2.61 p = .11; Decisiveness F(1, 195) = 3.69, p = .07; Discomfort with ambiguity F(1,195) = 0.17, p = .69; Closed-mindedness F(1, 195) = 1.80, p = .17).

### Correlations between the level of perceived stress and tolerance of ambiguity, stress from uncertainty, need for cognitive closure

Pearson’s two-tailed correlations between the level of perceived stress and all other measures are reported in Table 3.

**Table 3.** Correlation matrix for all analyzed measures.

	1	2	3	4	5	6	7
1. JSQ							
2. TFA	−.318*						
3. PRU: stress from uncertainty	.433**	−.467**					
4. NFC: preference for order	.209**	−.439**	.313**				
5. NFC: preference for predictability	.175*	−.444**	.311**	.657**			
6. NFC: decisiveness	−.075	−.252**	.083	.399**	.359**		
7. NFC: discomfort with ambiguity	.131*	−.372**	.288**	.734**	.668**	.452**	
8. NFC: closed-mindedness	.075	−.319**	.188**	.517**	.423**	.339**	.508**

* p ≤ .05

** p ≤ .01

Results indicated that work-related stress had a moderate negative correlation with tolerance of ambiguity and a moderate positive correlation with the level of stress for uncertainty. A weak positive correlation was present between work-related stress and three NFCS subscales, precisely the Preference for order, Preference for predictability, and Discomfort with ambiguity subscales.

Tolerance for ambiguity turned out to have a moderate negative correlation with all other measures, that is, stress from uncertainty and all NFCS subscales. The same correlation pattern emerged for uncertainty scores, which had a moderate positive correlation with all NFCS subscales except for the Decisiveness dimension. Finally, all NFCS subscales showed moderate-to-strong positive intercorrelations.

### Influence of tolerance for ambiguity, stress from uncertainty and need for cognitive closure on perceived stress

Demographic and professional predictors (gender, years of experience, specialty) were first entered in a hierarchical linear regression, following all other psychological predictors were entered (tolerance for ambiguity, stress from uncertainty, and all NFCS dimensions). Table 4 displays partial correlations, the standardized regression coefficients (ß), and R-square after entry of all independent variables.

**Table 4.** Hierarchical linear regression with perceived stress as the dependent variable and gender, years of experience, specialty (first block), tolerance of ambiguity, stress from uncertainty, and need for cognitive closure (second block) as independent variables: partial correlations, the standardized regression coefficients (ß), and R-square.

Predictor	ß	Partial correlation with perceived stress
1. gender	−.03 (n.s.)	−.02 (n.s.)
2. years of experience	−.14 (p < .05)	−.16 (p < .05)
3. medical specialty	.02 (n.s.)	.04 (n.s.)
4. TFA	−.17 (p < .05)	−.16 (p < .05)
5. PRU: stress from uncertainty	.34 (p < .001)	.32 (p < .001)
6. NFC: preference for order	.13 (n.s.)	.12 (n.s.)
7. NFC: preference for predictability	−.01 (n.s.)	−.01 (n.s.)
5. NFC: decisiveness	−.18 (p < .005)	−.17 (p < .005)
6. NFC: discomfort with ambiguity	.17 (p < .05)	.16 (p < .05)
7. NFC: closed-mindedness	−.02 (n.s.)	−.01 (n.s.)

First block (demographich/professional variables): R^2^ = .03; F(3,206) = 2.03, p = .11

After entering second block (psychological variables): R^2^ = .32; F(10,206) = 8.78, p < .001.

The regression model, including all independent variables, turned out to be statistically significant [R^2^ = 0.32; F(10,206) = 8.78, p < .001], indicating that 32% of the variation in perceived stress is accounted for by a linear combination of the predictors included in the regression model.

After entering the psychological predictors in the regression analysis, the variance accounted for in the level of perceived stress significantly improved, beyond that afforded by the demographic and professional variables. In particular, the strongest predictors of stress turned out to be the stress specifically related to uncertainty, that is, one score increase in stress from uncertainty leads to a .34 increase in perceived work-related stress. Results indicated that tolerance for ambiguity is a significant predictor of perceived stress, showing that an increase in ambiguity tolerance scale produced a .17 decrease in the level of stress. Two of the NFCS subscales were significant predictors of stress, specifically the Decisiveness subscale turned out to predict lower level stress, whereas the Discomfort with ambiguity subscale predicted higher stress.

These associations were not influenced by either gender or medical specialty. In contrast, the years of experience turned out to be a predictor of the level of perceived stress, meaning that one more year of experience leads to a decrease of .15 in the level of perceived stress.

## Discussion

### The association between tolerance for ambiguity, stress from uncertainty and need for cognitive closure and perceived stress

The present study aimed at investigating the association among work stress and three measures – tolerance of ambiguity, stress from uncertainty, and need for cognitive closure – that are distinct and peculiar, yet partially overlapping. The findings revealed that those individual characteristics account for a significant part of the work stress variance even when demographic and professional characteristics were controlled statistically.

In line with previous research [27], the present study suggested that the higher inability to tolerate ambiguity significantly predicts higher level of perceived work-related stress. Possibly, high ambiguity intolerant physicians try to handle ambiguous situations by applying a sort of dichotomous thinking (which is probably closer to the idea that medicine mainly deals with ‘black-and-white’ decisions), even though most of the medical decisions would require the ability to take into account, explore, and ‘tolerate’ the existence of multiple possibilities, which fall outside the black-and-white terms. This way of approaching ambiguity could make ambiguity intolerant physicians feel unsecure and less confident about their own competencies, thus experiencing high levels of stress.

As for the relationship between uncertainty and work-related stress, our findings are consistent with the literature [5,7,37], which found significant associations between stress from uncertainty and other indicators of work well-being, such as risk of burnout and work-related satisfaction [5]. Specifically, our results suggest that stress from uncertainty turned out to be the strongest predictor of work-related stress. Given its prevalent focus on a ‘future time’, the uncertainty intolerance mainly regards a feeling of uneasiness and discomfort with the anticipation of possible future consequences. It is likely that high uncertainty intolerant physicians feel particularly threatened by the possible future consequences of their own decisions. When confronted with a clinical situation whose consequences are not easy to predict – and this concerns most medical decision making – high uncertainty intolerant physicians may feel particularly worried and anxious about the implications of their decisions, thus resulting in a feeling of being ‘stuck’ in the uncertainty and unable to move forward [38].

As for the need for cognitive closure, our results indicated that two subscales (Discomfort with ambiguity and Decisiveness) could be considered good predictors for work-related stress. As far as the association between the discomfort with ambiguity and stress is concerned, we refer to the comments reported above (at the beginning of the discussion section) with regard to the tolerance for ambiguity. The relationship between decisiveness and work-related stress among physicians, which to our knowledge has not been demonstrated before, indicated that the less decisiveness the more the physicians perceived stress. It has been fiercely debated [39,40] what the Decisiveness scale exactly measures. Whereas decisiveness has been traditionally intended as the ‘need and motivation to make quick decisions’ (i.e., urgency strivings) [40], more recent research provided empirical support to the idea that decisiveness scale refers to the ‘ability’ to make such decisions [41,42]. This tendency to moving quickly toward a decision has been found to be negatively associated with anxiety and discomfort, thus indicating that it motivates going directly to a conclusion before any psychological distress related to uncertainty can be experienced [43]. Possibly, physicians with high decisiveness were those who really possessed the ability to hastily reach a solution without experiencing the discomfort that could derive from the unknown and uncertain.

### The association between demographic and professional characteristics and the level of perceived stress

In relation to demographic and professional characteristics, it turned out that the level of stress was affected neither by gender nor specialty. Concerning the association between gender and stress, the literature provided mixed evidence. Whereas some studies found that female physicians reported higher level of stress [44,45], others found no differences related to gender [29]. Probably, to disentangle this matter, it would be necessary to consider other variables – not frequently measured within this literature – concerning the female physicians’ family status or the relational support they receive [46]. It could be the case that these aspects weigh on women’s general evaluation of their level of stress or satisfaction.

As for the type of specialty, our results are consistent with most literature, which found no differences in terms of work-related stress and satisfaction across specialties [46–48]. However, a study by Leigh and colleagues [29] challenged these findings, since these authors reported a higher proportion of dissatisfied U.S. physicians among those practicing ‘procedural’ specialties as compared to those who were in ‘cognitive’ disciplines. The authors identified the possible causes of dissatisfaction in the different level of income and prestige within the two types of specialties. We believe that our results could not be compared to this study for two distinct reasons. First, the U.S. and the Italian health-care systems are so different that they can hardly be compared; Among others, the so-called ‘prestige’ of the different medical specialties and the corresponding income are not the same in the two countries. Additionally, in the study by Leigh and colleagues it was only measured the level of satisfaction and, despite the high negative correlation they found, stress and satisfaction could not be considered one the exact opposite of the other.

As far as the association between the years of practice and work-related stress, consistently with previous research [5,29] our results indicated that the more work experience, the less the physicians perceived work-related stress. Possibly, the greater clinical experience accumulated across time might have made physicians more self-confident and self-efficient about their own competencies, thus making them feeling to have control over the situation. Even though our study did not directly address the relationship between the years of experience and the level of tolerance for ambiguity in determining the level of stress, it would be possible to hypothesize that the level of perceived stress decreased in more expert physicians due to their increased ability to tolerate ambiguity. The present study allowed us to only compare differently skilled groups on the ambiguity tolerance. A prospective design could properly verify whether (and, if so, how) the individual ability to tolerate ambiguity modifies within the same group of physicians in relation to gradually gaining experience. This possible line of research could also contribute to clarify the nature of the ambiguity tolerance – intended as an incremental individual ability [27] – and, thus, open to a major introduction of tools to manage ambiguity and uncertainty in medical education settings.

### Limitations and strengths

The main limitation of the study, which is quite common to most individual differences literature, is the exclusive reliance on self-reported rating scales. Even though it permitted us to reach a large number of physicians, we are aware that the possibility to combine and integrate different measures of the constructs (e.g., direct observations or semi-structured interviews) could provide a more comprehensive and multi-faceted picture of the topic. Future research could address this point by combining different way of measuring the physicians’ personal characteristics that were investigated in the present study. A second limitation concerns the cross-sectional nature of the data, implying that causal relations are subject to interpretation. Theoretically, it could have been that higher levels of work-related stress caused a significant decrease in the individual ability to tolerate ambiguity and uncertainty, and not the opposite. Nevertheless, a number of authors have assigned a causal interpretation to the relationships between being ambiguity and uncertainty tolerant and various indicators of psychological distress [5]. Moreover, it needs to be underlined that the regression analysis indicates that only 32% of the variation in perceived stress is accounted for by a linear combination of the predictors included in the regression model. Hence, further research is also needed to examine how other physicians’ unmeasured characteristics, including both contextual (e.g., specific work conditions) and personal (e.g., individual coping style), might influence the level of perceived stress.

Despite these limitations, it is worth noting that our results were obtained among a large sample of physicians including a high number of medical specialties, which made the sample quite representative of Italian physicians overall, thus permitting a moderately safe generalization of results.

### Implications

Findings from the present study have some implications for medical education. Given the influence that physicians’ ability to tolerate ambiguity and uncertainty have on the level of perceived work-related stress, it would be worth paying particular attention to such abilities. Specifically, the assessment of those individual characteristics could be an important factor during the admission test to medical schools. Probably, taking into consideration, among others, also the individual ability to tolerate ambiguity and uncertainty could affect, in the long term, the physical and mental wellbeing of physicians and result, as a consequence, in a better quality of health care practice in ambiguous conditions.

Furthermore, medical education should directly address the ambiguity and uncertainty inherent in medicine. A number of studies suggested that ambiguity and uncertainty tolerance might change over time and experience [17,18,49,50], thus opening up the possibility to cultivate them in medical school. It would be crucial to introduce or to empower, where it has been already introduced, measurement [51] and educational tools that could track any pattern of growth in medical students’ ability to tolerate ambiguity and uncertainty. In this respect, we believe that any tools or strategies should focus on both the emotional and cognitive reactions that could be associated with ambiguity and uncertainty. Indeed, ambiguity and uncertainty by themselves may not be problematic, provided that physicians are able to manage them. On the contrary, the anxiety and the frustration produced by ambiguity may be detrimental and should receive specific attention in medical education. In this respect, we suggest that one possible way to help medical students to successfully deal with anxiety and stress deriving from ambiguity and uncertainty is to first become fully aware of their own cognitive and emotional processes caused by medical uncertainty. Once they are conscious of ‘how they think and feel’ in response to uncertainty, medical students may monitor these cognitive and emotional processes and, in case, try to adjust them. Awareness, monitoring, and control of one’s own mental processes have been usually considered as components of metacognition [52]. The metacognitive approach, which stresses the learners’ need for reflection upon the cognitive and emotional processes they activate when facing a task [53], has been shown to be a promising perspective in different kind of educational settings [54–56]. In fact, it promotes the ability to identify the relevant strategies to be applied in a specific situation and to self-regulate behavior accordingly [57,58]. The introduction of reflective practices could be an effective way for medical students to become aware, monitor and, in some cases, modify their own way of dealing with clinical ambiguity as well as the negative reactions that may accompany it.

## Conclusions

Ambiguity and uncertainty are inevitable aspects of medicine. Findings from the present study suggest that the physicians’ ability to tolerate them, together with the ability to make quick decisions (decisiveness), may influence the level of work-related stress they perceive. Given their relevance in affecting physicians’ well-being, ambiguity and uncertainty should be directly addressed by medical education, which should introduce specific tools or strategies to cultivate the ability to successfully dealing with ambiguity and uncertainty.
